# A worldwide correlation of lactase persistence phenotype and genotypes

**DOI:** 10.1186/1471-2148-10-36

**Published:** 2010-02-09

**Authors:** Yuval Itan, Bryony L Jones, Catherine JE Ingram, Dallas M Swallow, Mark G Thomas

**Affiliations:** 1 Research Department of Genetics, Evolution and Environment, University College London, London WC1E 6BT, UK; 2 CoMPLEX (Centre for Mathematics & Physics in the Life Sciences and Experimental Biology), University College London, London WC1E 6BT, UK; 3 AHRC Centre for the Evolution of Cultural Diversity, Institute of Archaeology, University College London, 31-34 Gordon Square, London WC1H 0PY, UK

## Abstract

**Background:**

The ability of adult humans to digest the milk sugar lactose - lactase persistence - is a dominant Mendelian trait that has been a subject of extensive genetic, medical and evolutionary research. Lactase persistence is common in people of European ancestry as well as some African, Middle Eastern and Southern Asian groups, but is rare or absent elsewhere in the world. The recent identification of independent nucleotide changes that are strongly associated with lactase persistence in different populations worldwide has led to the possibility of genetic tests for the trait. However, it is highly unlikely that all lactase persistence-associated variants are known. Using an extensive database of lactase persistence phenotype frequencies, together with information on how those data were collected and data on the frequencies of lactase persistence variants, we present a global summary of the extent to which current genetic knowledge can explain lactase persistence phenotype frequency.

**Results:**

We used surface interpolation of Old World lactase persistence genotype and phenotype frequency estimates obtained from all available literature and perform a comparison between predicted and observed trait frequencies in continuous space. By accommodating additional data on sample numbers and known false negative and false positive rates for the various lactase persistence phenotype tests (blood glucose and breath hydrogen), we also apply a Monte Carlo method to estimate the probability that known lactase persistence-associated allele frequencies can explain observed trait frequencies in different regions.

**Conclusion:**

Lactase persistence genotype data is currently insufficient to explain lactase persistence phenotype frequency in much of western and southern Africa, southeastern Europe, the Middle East and parts of central and southern Asia. We suggest that further studies of genetic variation in these regions should reveal additional nucleotide variants that are associated with lactase persistence.

## Background

**Figure 1:**
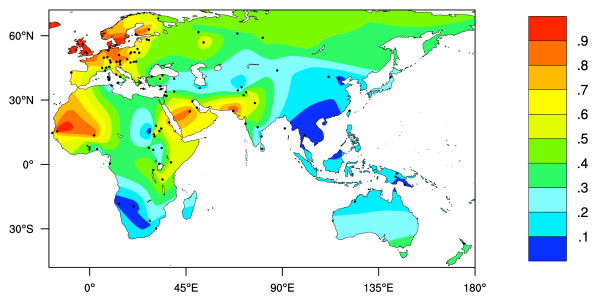
**Interpolated map of Old World LP phenotype frequencies**. Dots represent collection locations. Colours and colour key show the frequencies of the LP phenotype estimated by surface interpolation.

An estimated 65% of human adults (and most adult mammals) downregulate the production of intestinal lactase after weaning. Lactase is necessary for the digestion of lactose, the main carbohydrate in milk [1], and without it, milk consumption can lead to bloating, flatulence, cramps and nausea [2]. Continued production of lactase throughout adult life (lactase persistence, LP) is a genetically determined trait and is found at moderate to high frequencies in Europeans and some African, Middle Eastern and Southern Asian populations (see Additional File 1 and Figure 1).

The most frequently used non-invasive methods for identifying the presence of intestinal lactase are based upon detecting digestion products of lactose produced by the subject (Blood Glucose, BG) or gut bacteria (Breath Hydrogen, BH). For both methods a lactose load is administered to the subject following an overnight fast. In individuals producing lactase this leads to a detectable increase in blood glucose. In individuals who are not producing lactase, the undigested lactose will pass into the colon where it is fermented by various gut bacteria, producing fatty acids and various gases, particularly hydrogen. Hydrogen passes through the blood into the lungs and so can be detected in the breath using a portable hydrogen analyser. Both the BG and the BH tests have asymmetric type I and type II error rates. Thus any study seeking association between a particular polymorphism and LP should take these error rates into account. In addition it should be noted that while in most cases the presence/absence of intestinal lactase in an adult is likely to be genetically determined, the loss of lactase can also be caused by gut trauma such as gastroenteritis [3-6]. Other non-invasive methods for detecting the presence/absence of lactase include assaying for urine galactose and detecting metabolites of Carbon-14-labelled lactose. These methods are rarely used today. The most reliable method is intestinal biopsy, which provides a direct determination of intestinal lactase activity. However, this procedure is very rarely used for diagnosing healthy individuals because of its invasive nature [7].

With the recent discovery of nucleotide changes associated with LP comes the prospect of direct genetic tests for the trait [8-10]. However, it has become clear that there are multiple, independently derived LP-associated alleles with different geographical distributions [1,8,11,12]. LP is particularly common in Europe and certain African and Middle Eastern groups. As a consequence these are the regions where most genetic studies have been focused and all currently known LP alleles have been identified [7,11,12]. The first allelic variant that was shown to be strongly associated with increased lactase activity is a C>T change 13,910 bases upstream of the LCT gene in the 13^th ^intron of the MCM6 gene [13]. Functional studies have indicated that this change may affect lactase gene promoter activity and increase the production of lactase-phlorizin hydrolase mRNA in the intestinal mucosa [14-17] but, as with all LP-associated variants, there remains the possibility that linkage to as yet unknown causative nucleotide changes may explain observed associations. Haplotype length conservation [18], linked microsatellite variation [19] and ancient DNA analysis from early European farmers [20] later confirmed that this allele has a recent evolutionary origin and had been the subject of strong positive natural selection. Furthermore, a simulation model of the origins and evolution of lactase persistence and dairying in Europe has inferred that natural selection started to act on an initially small number of lactase persistent dairyers around 7,500 BP in a region between Central Europe and the northern Balkans, possibly in association with the Linearbandkeramik culture [21]. Another simulation study has inferred that it is likely that lactase persistence selective advantage was not constant over Europe, and that demography was a significant element in the evolution and spread of European lactase persistence [22].

However, the presence of this allele could not explain the frequency of LP in most African populations [8]. Further studies identified three additional variants that are strongly associated with LP in some African and Middle Eastern populations and/or have evidence of function, all are upstream of the *LCT *gene in the 13^th ^intron of the *MCM6 *gene: -13,907*G, -13,915*G and -14,010*C [11,12,23,24]. Where data were sufficient, some of these alleles also showed genetic signatures of a recent origin and strong positive natural selection [12,23].

Although at least four strong candidate causative alleles have been identified, only a small number of populations have been studied, and those are confined to Europe, Africa and the Middle East. It is therefore unlikely that all LP-associated or LP-causing alleles are currently known. As a consequence, genetic tests based on current knowledge would underestimate the frequency of LP in most world populations. As part of the first study to seek a genetic explanation for the distribution of LP in Africa [8], a statistical procedure (*GenoPheno*) was developed to test if the frequency of an LP-associated allele could explain reported LP frequency in ethnically matched populations. Crucially, this statistical procedure was designed to account for sampling errors and the asymmetric type I and type II error rates associated with different phenotype tests (BH and BG).

In this study we have sought to extend this approach to the whole of the Old World. However, while there is a rich literature on the frequencies of LP in different geographic regions [1] and a growing body of publications reporting the frequencies of candidate LP-causing alleles, in most cases the genetic and phenotypic data are not from the same people and often not of closely neighbouring groups. Thus, characterization of the extent to which LP frequency can be explained by current knowledge of LP-associated genotype frequencies is limited to populations where both data types are available. To overcome this problem we performed surface interpolation of various data categories (genetic, phenotypic, sample numbers, phenotype tests used and their associated error rates) and applied the statistical procedures described on a fine grid covering the Old World landmass. This has allowed us to identify regions where reported LP-associated allele frequencies are insufficient to explain the presence of LP. These regions should be good candidates for future genotype/phenotype studies.

## Methods

### Data

Our global LP phenotype dataset consists of 112 locations [1] (see Additional File 1). These data were carefully selected from a large literature on LP frequencies so as to remove data collected from (1) children, (2) patients selected for likely lactose intolerance, (3) family members, and (4) people with twentieth/twenty-first century immigrant status. Genotype data was obtained for 132 locations where the frequency of the -13,910 C>T allele had been estimated [7,8,11,18,25-27], and from 61 locations where the frequency of all 4 currently known LP associated allelic variants had been estimated [12,23,26,28] (see Additional File 2). These data were carefully selected from a large literature on LP frequencies so as to remove data collected from (1) patients selected for likely lactose intolerance, (2) family members, and (3) people with twentieth/twenty-first century immigrant status. Where there was more than one dataset for a particular location (for either genotype or phenotype data), a weighted average frequency was calculated. The type I and type II error rates used were 8.621% and 6.849%, respectively, for BG and 6.818% and 4.167%, respectively, for BH [8]. Predicted LP frequencies, from the LP genotype frequencies, were calculated by assuming *Hardy-Weinberg *equilibrium and dominance (see Additional File 2).

The geographic space explored was from longitude -19 to 180, and from latitude -48 to 72.

### Surface Interpolation

To estimate the distribution of LP and LP-associated allele frequencies in continuous space, from irregularly spaced data, surface interpolation was performed using the *Natural Neighbour *algorithm [29,30], as implemented in the *PyNGL *module of the Python programming language [31-33]. Briefly, this algorithm first divides a 2-dimentional space into polygons according to the locations of the observed data points, then estimates the value at locations for which data is absent by weighting each of the neighbouring locations by their relative overlap.

### Quantitative Difference Correlation Analysis

We also performed an analysis to quantify the difference between phenotype frequency and predicted phenotype frequency based on the frequency of LP-associated alleles. As above, we assumed *Hardy-Weinberg *equilibrium and performed surface interpolation using the data provided in the tables of Additional Files 1 and 2. We then subtracted the surface representing expected frequencies from that representing observed LP frequencies. Maps were plotted using *PyNGL *[33]. The code for the program is available from the authors.

### *GenoPheno *Correlation Analysis

To identify regions where LP-associated allele frequencies are insufficient to explain observed LP incidence we applied the Monte Carlo based statistical test *GenoPheno *[8] to each cell in a 198 (west-east) by 119 (south-north) grid of covering the Old World. For each cell it was necessary to provide information on LP-associated allele frequencies and LP incidence (see above) as well as on sample numbers used for each data type and type I and type II error rates for the LP phenotype tests used. These parameters were estimated by surface interpolating values from genetic and phenotypic studies to provide 6 surface interpolated 'layers' of information. The code for the program is available from the authors.

### Similar Populations at Similar Regions Analysis

To demonstrate LP specific genotype-phenotype correlation analysis without interpolation (Table 1) we have performed quantitative difference and GenoPheno tests (as described above) on phenotype and genotype data (from the tables of Additional Files 1 and 2, respectively) where: (1) the ethnic groups are similar, and (2) the country/region is similar and a deviation of maximum 3 degrees between the two data points.

**Table 1:** The GenoPheno correlation for similar ethnic groups at similar regions

**Population**			**Lactase persistence genotype data**				**Lactase persistence phenotype data**				**Genotype-phenotype correlation**	
			
**Continent**	**Country**	**Population**	**N**	**Sum of LP-associated alleles frequency**	**Predicted LP freq.**	**Ref.**	**N**	**Pheno. Freq.**	**Testing method**	**Ref.**	***GenoPheno *P-value**	**Genotype - phenotype quantitative difference**
			
Africa	Nigeria	Yoruba	50	0.00	0.00	[18]	48	0.17	BG	[43]	0.5670	0.17
Africa	Senegal	Wolof	118	0.00	0.00	[24]	53	0.51	BG	[44]	<0.00005	0.51
Africa	Sudan	Beni Amer	162	0.26	0.45	[24]	40	0.88	BH	[45]	<0.00005	0.42
Africa	Uganda	Bantu	44	0.00	0.00	[8]	17	0.06	BG	[46]	0.7552	0.06
Asia	China	Kazakh	94	0.00	0.00	[47]	195	0.24	BH	[48]	0.0026	0.24
Asia	India	Indian	68	0.07	0.14	[7]	38	0.00	BIOPSY	[49]	0.0024	-0.14
Asia	Japan	Japanese	62	0.00	0.00	[18]	40	0.28	BG	[50]	0.0432	0.28
Asia	North India	Northern Indian	128	0.33	0.55	[7]	136	0.55	BG	[51,52]	1.0000	0.00
Asia	Russia	Komi	20	0.15	0.28	[23]	56	0.38	BG	[53]	0.9248	0.10
Europe	Finland	Finns	1876	0.58	0.82	[23]	638	0.83	BIOPSY	[54]	0.3686	0.01
Europe	Greece	Greek	100	0.09	0.17	[40]	200	0.25	BH	[55]	0.8136	0.08
Europe	Hungary	Hungarian	110	0.62	0.86	[56]	262	0.59	BH	[57]	<0.00005	-0.26
Europe	Ireland	Irish	65	0.95	1.00	[8]	50	0.96	BG	[58]	0.6014	-0.04
Europe	Italy	Sardinian	153	0.07	0.14	[40]	47	0.15	BH	[59]	0.5530	0.01
Europe	Italy	North Italian	28	0.36	0.59	[18]	208	0.49	BH	[60]	0.3348	-0.10
Europe	Italy	Central Italian	98	0.11	0.21	[40]	65	0.82	BG	[61]	<0.00005	0.61
Europe	Italy	Southern Italian	189	0.08	0.15	[40]	99	0.46	BH	[62]	<0.00005	0.31
Near/Middle East	Afghanistan	Tadjik	98	0.30	0.51	[7]	79	0.18	BG	[63]	<0.00005	-0.33
Near/Middle East	Afghanistan	Pashtu	16	0.10	0.19	[18]	71	0.21	BG	[63]	0.3588	0.02
Near/Middle East	Iran	Iranian	42	0.10	0.19	[23]	21	0.14	BG	[64]	0.4154	-0.05
Near/Middle East	Israel	Arabs	160	0.05	0.10	[24]	67	0.19	BG	[65]	0.8966	0.10
Near/Middle East	Jordan	Jordanian	112	0.11	0.20	[23]	162	0.76	BH	[66]	<0.00005	0.55
Near/Middle East	Pakistan	Balochi	50	0.00	0.00	[18]	32	0.38	BH	[67]	0.0036	0.38
Near/Middle East	Saudi Arabia	Bedouin	94	0.48	0.73	[23]	21	0.81	BH	[68]	0.5166	0.08
Near/Middle East	Turkey	Turks	98	0.03	0.06	[8]	126	0.30	BH	[69]	0.0076	0.24

*GenoPheno *was used to calculate the P-value for lack of correlation between the predicted phenotype (based on all 4 known LP-associated alleles) and the observed phenotype at locations where the ethnic groups were similar, and so expected to have a good genotype-phenotype correlation. Unlike figures 5 and 6, interpolation was not used here, but rather observed points analyses.

### Sample Density Analysis

To indicate regions where genotype and phenotype sampling is sparse we used a 2-dimensional kernel density estimation [34,35], as implemented in the *KernSmooth *package of the R statistical programming environment [36]. We used an isotropic kernel with a bandwidth equal to half of the average nearest neighbour distance (ANND) between sample points, to ensure that >95% of each Gaussian sampling region will be within the ANND.

## Results

### Interpolated LP Phenotype Frequencies

Figure 1 shows an interpolated map of the frequencies of LP based on phenotype tests (also see Additional File 1, [1]). Although this map should provide a reasonable representation of frequencies in Europe and western Asia, it should be noted that (1) data is sparse at eastern and northern Asia, Indonesia, Melanesia, Australia and Polynesia, and (2) in Africa and the Middle East it is often the case that populations living in close proximity to each other have dramatically different LP frequencies, depending to an extent on traditional subsistence strategies [1].

### Interpolated Predicted LP Phenotype Frequencies

**Figure 2:**
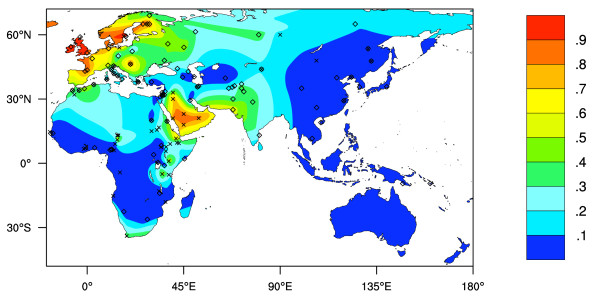
**Predicted Old World LP phenotype frequencies based on LP-associated allele frequencies**. LP frequency prediction assumes *Hardy-Weinberg *equilibrium and dominance. Crosses represent collection locations where all 4 currently known LP-associated alleles were genotyped, and diamonds represent collection locations where the only data on the -13,910 C>T allele is available. Colour key shows the predicted LP phenotype frequencies estimated by surface interpolation.

Figure 2 shows an interpolated map of the frequencies of LP predicted by all 4 currently known LP associated allelic variants, based on genotyping tests (see Additional File 2, [7,8,11,18,25-27]). As with the phenotype data, the genotype data is sparse in eastern and northern Asia, Indonesia, Melanesia, Australia and Polynesia.

**Figure 3:**
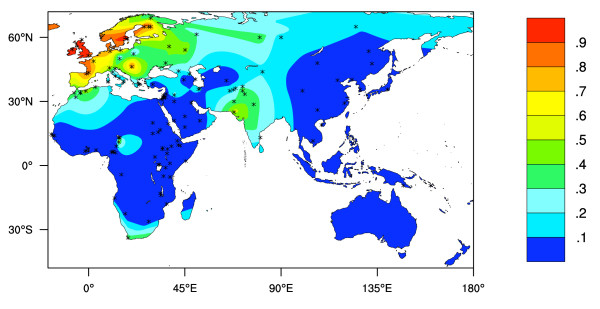
**Predicted Old World LP phenotype frequencies based on -13,910 C>T allele frequency data only**. LP frequency prediction assumes *Hardy-Weinberg *equilibrium and dominance. Stars represent collection locations. Colour key shows the predicted LP phenotype frequencies estimated by surface interpolation.

Figure 3 shows an interpolated map of the frequencies of LP predicted by the -13,910 C>T allele data only (see Additional File 2, [7,8,11,18,25-27]).

**Figure 4:**
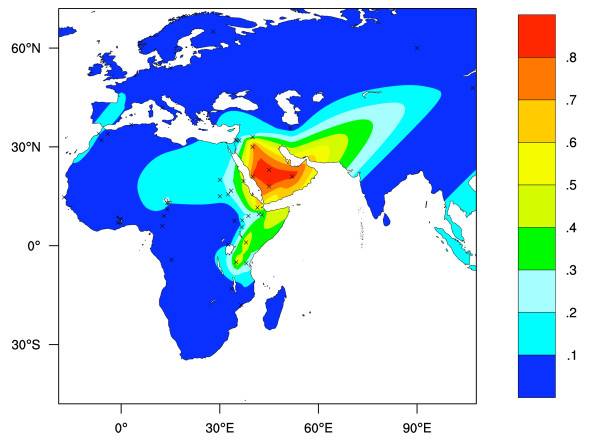
**Predicted Old World LP phenotype frequencies based on frequency data for the currently known LP associated allelic variants, excluding the -13,910 C>T allele**. LP frequency prediction assumes *Hardy-Weinberg *equilibrium and dominance. Crosses represent collection locations. Colour key shows the predicted LP phenotype frequencies estimated by surface interpolation.

Figure 4 shows an interpolated map of the frequencies of LP predicted by the 3 currently known LP associated allelic variants, excluding the -13,910 C>T allele (see Additional File 2, [12,23,26,28]). While this map provides a reasonable representation of the frequencies of the 3 LP associated allelic variants in eastern Africa and the Middle East, data from the rest of the world is sparse.

### LP Genotype-Phenotype Correlations

**Figure 5:**
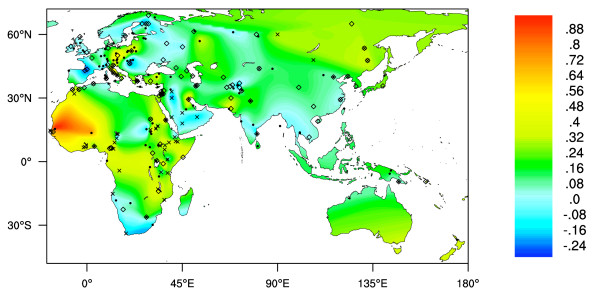
**Old World LP genotype-phenotype correlation, obtained by calculating the quantitative difference between observed LP phenotype frequency and that predicted using frequency data on all 4 LP-associated alleles**. Positive and negative values represent cases of LP-correlated genotype under- and over-predicting the LP phenotype, respectively. Dots represent LP phenotype collection locations, crosses represent data collection locations for all currently known 4 LP-correlated alleles, and diamonds represent -13,910 C>T only data collection locations. Colour key shows the values of the predicted LP phenotype frequencies (Figure 2) subtracted from the observed LP phenotype frequencies (Figure 1).

Figure 5 shows the quantitative difference between observed phenotype frequency and predicted phenotype frequency based on the frequency of 4 LP-associated alleles. This map was obtained by subtracting the surface shown in Figure 2 from that shown in Figure 1. It represents the extent to which current knowledge of the frequencies various LP-associated alleles explains the distribution of the LP trait. In many cases sample numbers used to obtain molecular and phenotype data were small. Additionally, phenotype testing error rates are appreciable. It is therefore possible that, for some regions, where the discrepancies between predicted and observed LP frequencies are high, such differences can be explained by sampling and testing errors alone.

**Figure 6:**
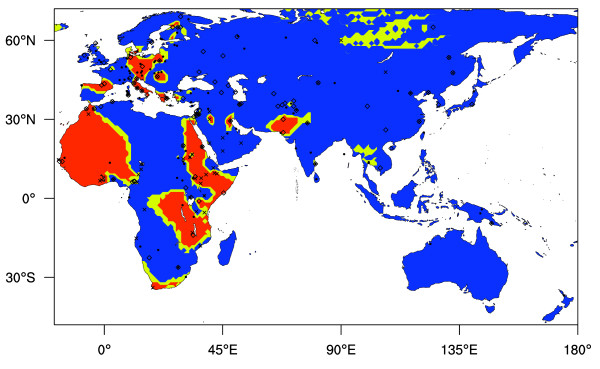
**Old World LP genotype-phenotype correlation, obtained by the *GenoPheno *Monte Carlo test**. Dots represent LP phenotype data collection locations, crosses represent data collection locations for all currently known 4 LP-correlated alleles, and diamonds represent collection locations for data on -13,910 C>T only. Colour key shows the *p *value obtained by the *GenoPheno *test. Red colour represents values of *p *< 0.01, indicating a highly significant lack of correlation, yellow colour represents values of 0.01 ≤ *p *< 0.05, indicating a significant lack of correlation, and blue colour represents values of *p *≥ 0.05, indicating no significant lack of correlation.

To account for the sampling and testing errors, we have applied the Monte Carlo based statistical test *GenoPheno *[8] to the surfaces presented in Figures 1 and 2. Performing this test also requires data on sample numbers and error rates, for which we generated interpolated surfaces by applying the same reasoning as we have to LP frequencies. By applying the *GenoPheno *test to 23,562 locations on a 198 by 119 cell grid we obtain the surface presented on Figure 6. These p-values approximate the probability of the observed genotype and phenotype data under the null hypothesis that the LP-associated alleles and phenotyping errors alone account for the observed LP frequency.

### Online Resource For LP Phenotype and Genotype Data and Associated Mapping

The LP phenotype and genotype datasets are available at the GLAD (Global Lactase persistence Association Database) web site http://www.ucl.ac.uk/mace-lab/GLAD/. These datasets will be updated every three months, and the maps corresponding to Figures 1 to 6 of this article will be regenerated from those updated databases. The web pages contain guidelines for what we consider "appropriate" LP data (as described at the Methods section). We encourage readers to send us new LP genotype and phenotype data whenever it is available.

## Discussion

In this study we have identified regions where the current data on LP-associated allele frequencies is insufficient to explain the estimated LP phenotype frequencies, by surface interpolating LP genotype and phenotype data. Our analyses also indicate regions where genotypic or phenotypic data is sparse or non-existent (see also the maps of Additional Files 3, 4, and 5). Data collection from these regions is likely to be of value in developing a fuller understanding of the distribution and evolution of LP. We suggest that regions where LP-associated genotypes are under-predicting LP are good candidates for further genetic studies.

We accept that surface interpolation can give misleading results when data are sparse and so urge caution in interpreting our results for such regions. The data used and maps generated will be regularly updated via the GLAD website http://www.ucl.ac.uk/mace-lab/GLAD/. We therefore expect that problems caused by interpolation over regions with sparse data will diminish as more data become available. However, to indicate regions where sampling is sparse we have generated three extra maps (Additional Files 3, 4, and 5) showing the density of sample sites for phenotypic data, for sites where -13,910*T allele data is available, and for sites where data on all 4 LP-associated alleles is available, respectively. These maps were generated using 2-dimentional-kernel density estimation [34-36] with a kernel bandwidth equal to half of the average nearest neighbour distance between sample sites.

While on a broad scale most regions of the Old World have been sampled for the -13,910*T allele, data on frequencies of the other three LP-associated alleles is localised mainly to Africa and the Middle East. It is likely that further studies will identify appreciable frequencies of the -13,907*G, -13,915*G or -14,010*C alleles, or reveal new LP-associated alleles, in other regions. To illustrate how data on the three non -13,910*T alleles have added to our knowledge of the genetics of LP, we have generated two additional maps: one is showing the correlation between the LP phenotype and the LP phenotype predicted by data where all 4 LP-associated alleles were sequenced (Additional File 6), and the second is showing the correlation between the LP phenotype and the LP phenotype predicted by data on the -13,910*T allele alone (Additional File 7). We then subtracted the surfaces of these two figures (Additional File 8).

Our analysis indicated a few regions (including Arabia and the Basque region) where the LP-associated allele frequency appears to over-predict LP phenotype frequency. If we assume that all four LP-associated alleles considered here are causative of the trait, or very tightly linked to causative variants, then it is likely that over-prediction is a result of population sampling problems. For example, the pastoralist Bedouin in Saudi Arabia have high frequencies of LP, while non-Bedouin Arabs from the same region typically have lower frequencies [37]. Alternatively, over-prediction may to an extent arise through post weaning non-genetic causes of LP, such as secondary lactose intolerance caused by gut trauma [3-6]. To an extent these problems of matching population groups from the same geographic regions applies to our whole analysis. However, it is notable from Figure 5 that where a lack of correspondence between LP phenotype and predicted phenotype frequencies occurs, it is usually under-prediction (maximum = 0.94; for West Africa), while over-prediction is rare and considerably smaller in scale (maximum = 0.29; for the Basque region). In some cases it was possible to identify genotype and phenotype data originating from the same populations or ethnic groups. For such populations we also performed a separate *GenoPheno *analysis (see Table 1, [8]). We note that the large discrepancies between LP phenotype frequencies and those predicted from allele frequency data, from this analysis of 'matched' populations, mostly occur in the same regions as where large discrepancies are indicated from the interpolation analysis (see Figure 5). This illustrates that - at a broad scale - the interpolation method that we have employed provides a reasonable approximation of where genotypic data are unable to explain the observed frequency of the LP phenotype, despite much of the data we use originating from different ethnic groups with different subsistence strategies [38,39].

By applying the *GenoPheno *statistical procedure to interpolated layers of phenotype and genotype associated data (Figure 6), we have identified west and parts of east Africa, eastern Europe, and parts of western, central, and southern Asia as potential targets for further genetic studies. An absence of data for the -13,907*G, -13,915*G and -14,010*C alleles in many of these regions may partly explain under-prediction (Figure 5 and Additional Files 6, 7, and 8). Previous studies have noted the possibility of under-prediction in eastern Europe and proposed the presence of alleles other than -13,910*T [40,41]. The population sampling problems described above may also explain the under-prediction we infer in parts of southern Asia, as in each of these regions, the locations where phenotype and genotype data were obtained are mostly well separated. This population data-matching problem is, however, unlikely to explain the lack of correspondence between LP and allele frequency-based predicted LP frequencies in the region around Pakistan and Afghanistan, as well as in west Africa and Italy. Further genetic studies in these regions should prove informative.

## Conclusion

In this study we have demonstrated that lactase persistence genotype data is currently insufficient to explain lactase persistence phenotype frequency in western and eastern Africa and several other Old World regions. The identification of additional LP-associated or LP-causative alleles, especially in these regions, will help not only in developing a better understanding of the evolution of LP but also in elucidating the physiological mechanisms that underlie the trait. The interpolation and mapping approach that we have applied in this study may also be of value in studying the underlying genetic basis and evolution of other phenotypic variation that impacts on human health, such as the distribution of functional variation in drug metabolising enzymes [42].

## Authors' contributions

YI and MGT initiated and designed the study. YI performed the analyses and the programming routines. MGT contributed biological, statistical, and anthropological expertise. DMS contributed lactase persistence genotype and phenotype expertise. YI, DMS, BLJ, and CJEI contributed to collating the data and editing the tables. YI and MGT wrote the article. All authors contributed in revising the article. All authors read and approved the final manuscript.

## Supplementary Material

Additional file 1 **A table of the lactase persistence phenotype frequencies**. Columns show location (continent, country, longitude and latitude), population group, number of individuals tested, frequency of lactase persistent individuals, LP test method, and the primary source reference. The Americas were excluded from the table due to paucity of data. Other reasons for data exclusion were: recent immigrant populations, children (under 12 years old), or biased individuals selection criteria (such as individuals reported being lactase non persistent or related individuals). Wherever only country name was available, location was determined by the capital city or the estimated central point of the country. Click here for file

Additional file 2 **A table of the lactase persistence associated allele frequencies**. Columns show location (continent, country, longitude and latitude), population group, number of individuals tested, frequency of -13910*T, -13,907*G, -13,915*G and -14,010*C LP-associated alleles, the sum of all LP-associated alleles, predicted lactase persistence frequency, and the primary literature and own data source. Data taken from SNP typing tests (where only -13,910*T is shown) or from resequencing. The Americas were excluded from the table due to paucity of data. The predicted lactase persistence frequency was calculated by assuming *Hardy-Weinberg *equilibrium and dominance using the sum of the all available LP-associated alleles at a specific location. Wherever only country name was available, location was determined by the capital city or the estimated central point of the country. It should be noted that the collection location for the Indian and North Indian genotype data was Singapore. As an exception, we placed these data in the location of the ancestral population because of lack of genetic data from India. Click here for file

Additional file 3 A map of the density of sample sites for phenotypic data. Click here for file

Additional file 4 A map of the density of sample sites where 13,910*T allele data is available. Click here for file

Additional file 5 A map of the density of sample sites where data of all 4 LP-associated alleles is available. Click here for file

Additional file 6 **Africa and Middle East LP genotype-phenotype correlation, obtained by calculating the quantitative difference between observed phenotype frequency and predicted phenotype frequency based on locations where only fully sequenced data of all 4-LP associated alleles was available**. Positive and negative values represent cases of LP-correlated genotype under- and over-predicting the LP phenotype, respectively. Dots represent LP phenotype collection locations, crosses represent data collection locations for all currently known 4 LP-correlated alleles. Colour key shows the values of the predicted LP phenotype frequencies (Figure 4) subtracted from the observed LP phenotype frequencies (Figure 1). The Asia-Pacific data was not analysed since 4 alleles data in these regions is very sparse, and fully sequenced data for western and northern Europe is also sparse. Click here for file

Additional file 7 Old World LP genotype-phenotype correlation, obtained by calculating the quantitative difference between observed phenotype frequency and predicted phenotype frequency based on -13,910*T allele data only. Positive and negative values represent cases of LP-correlated genotype under- and over-predicting the LP phenotype, respectively. Dots represent LP phenotype collection locations, crosses represent data collection locations for the 13,910*T allele obtained from fully sequenced data, and diamonds represent -13,910 C>T only data collection locations. Colour key shows the values of the predicted LP phenotype frequencies predicted by -13,910*T allele data only subtracted from the observed LP phenotype frequencies. Click here for file

Additional file 8 The difference between the maps of Additional Files 6 and 7, demonstrating the additional knowledge acquired by the 3 additional LP-associated alleles (other than the -13,910*T allele). The Asia-Pacific data was not analysed since 4 alleles data in these regions is very sparse. Click here for file
