# Supplementary figures and images for: A worldwide correlation of lactase persistence phenotype and genotypes
Source: BMC Evol Biol. 2010 Feb 9;10:36. doi: 10.1186/1471-2148-10-36 (PMC2834688; doi:10.1186/1471-2148-10-36)

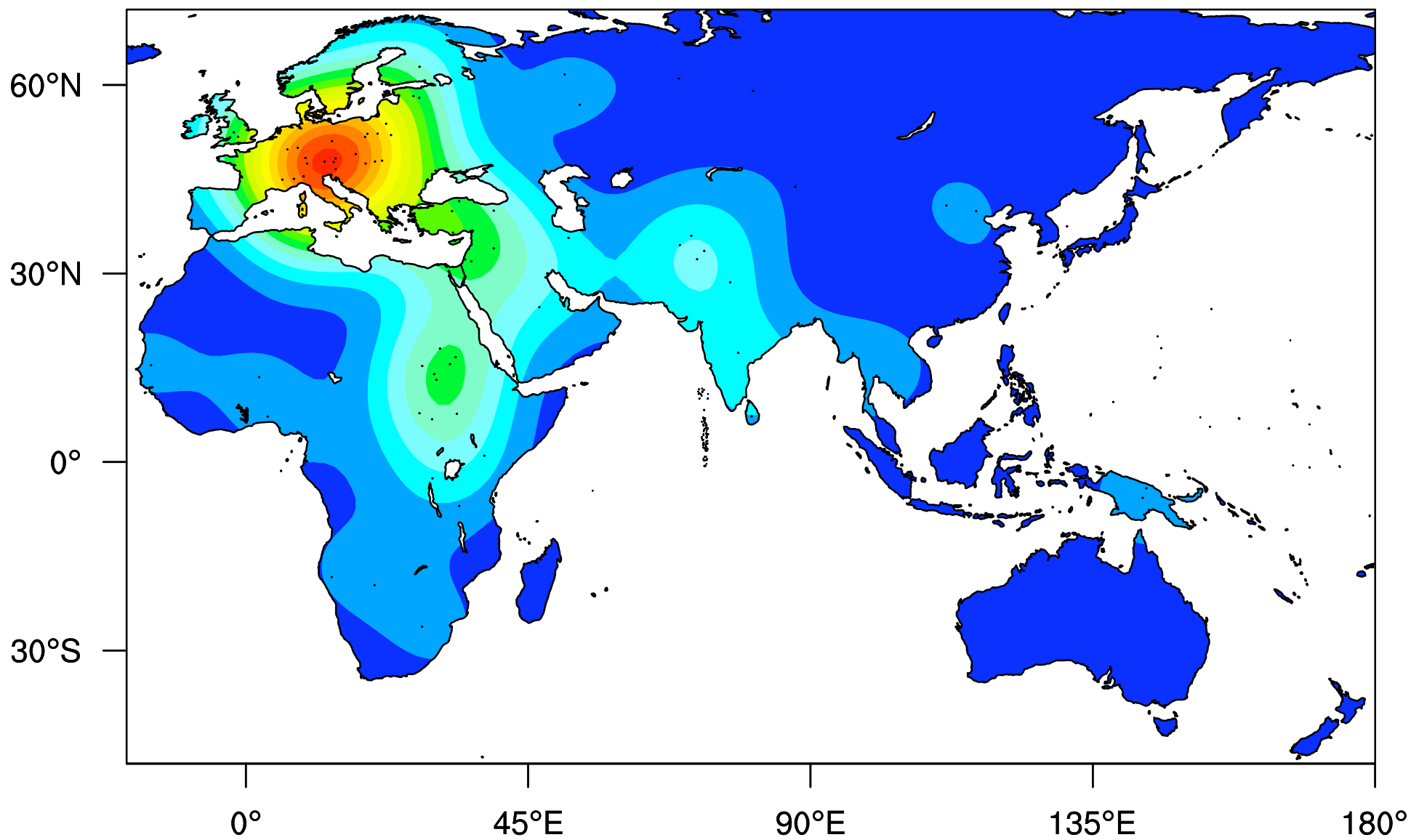

Supplement: Additional file 3 — A map of the density of sample sites for phenotypic data. [file 1471-2148-10-36-S3.PDF]

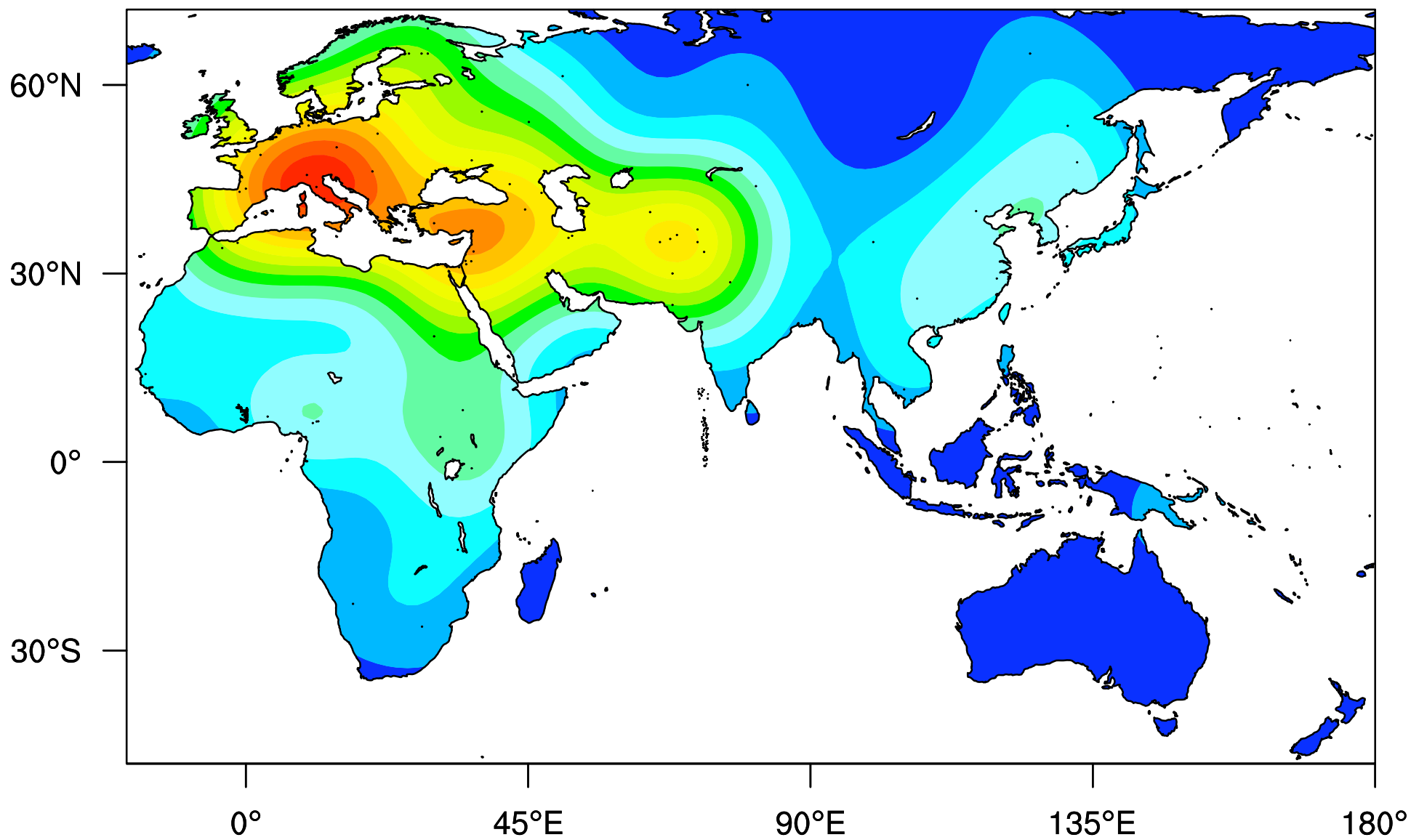

Supplement: Additional file 4 — A map of the density of sample sites where 13,910*T allele data is available. [file 1471-2148-10-36-S4.PDF]

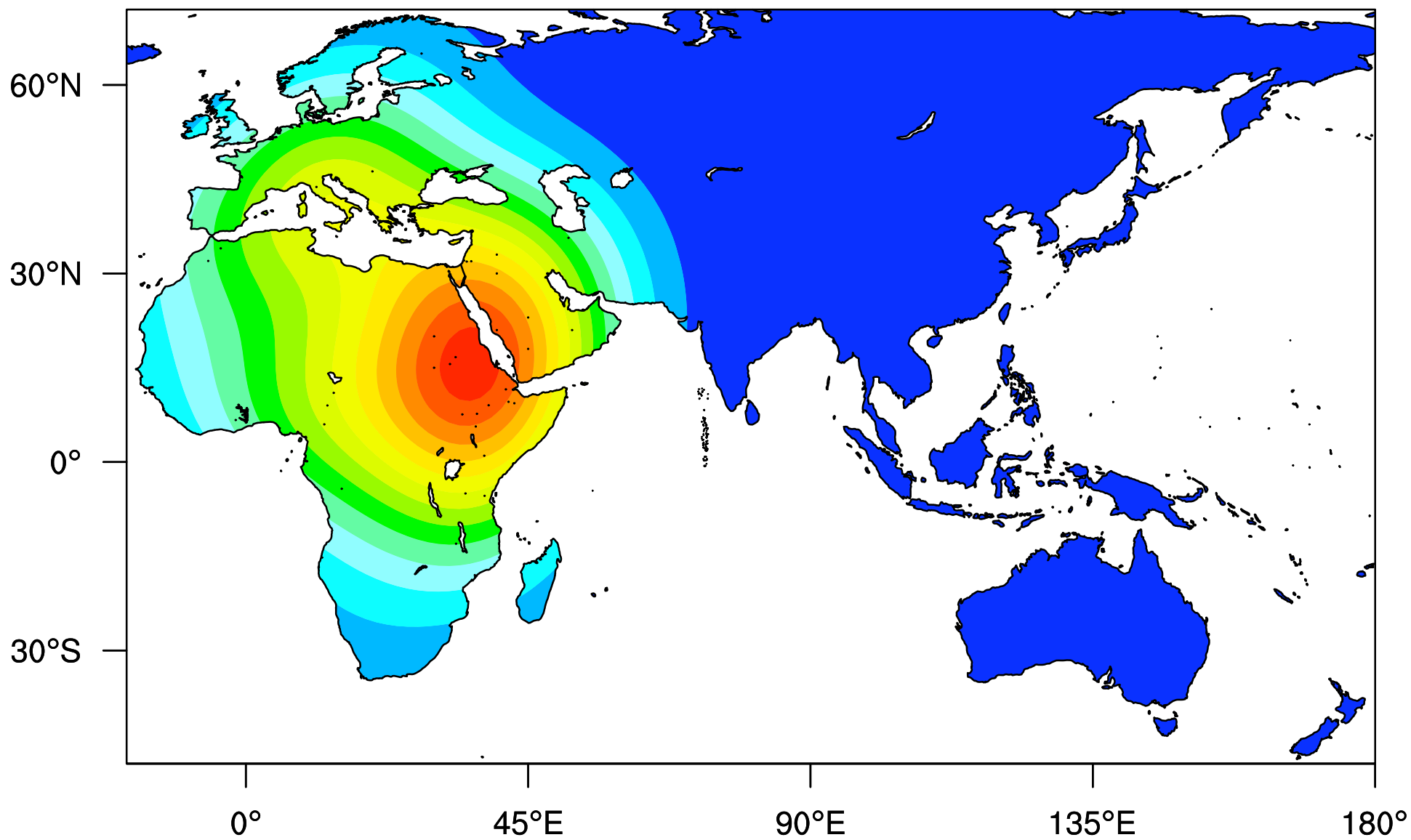

Supplement: Additional file 5 — A map of the density of sample sites where data of all 4 LP-associated alleles is available. [file 1471-2148-10-36-S5.PDF]

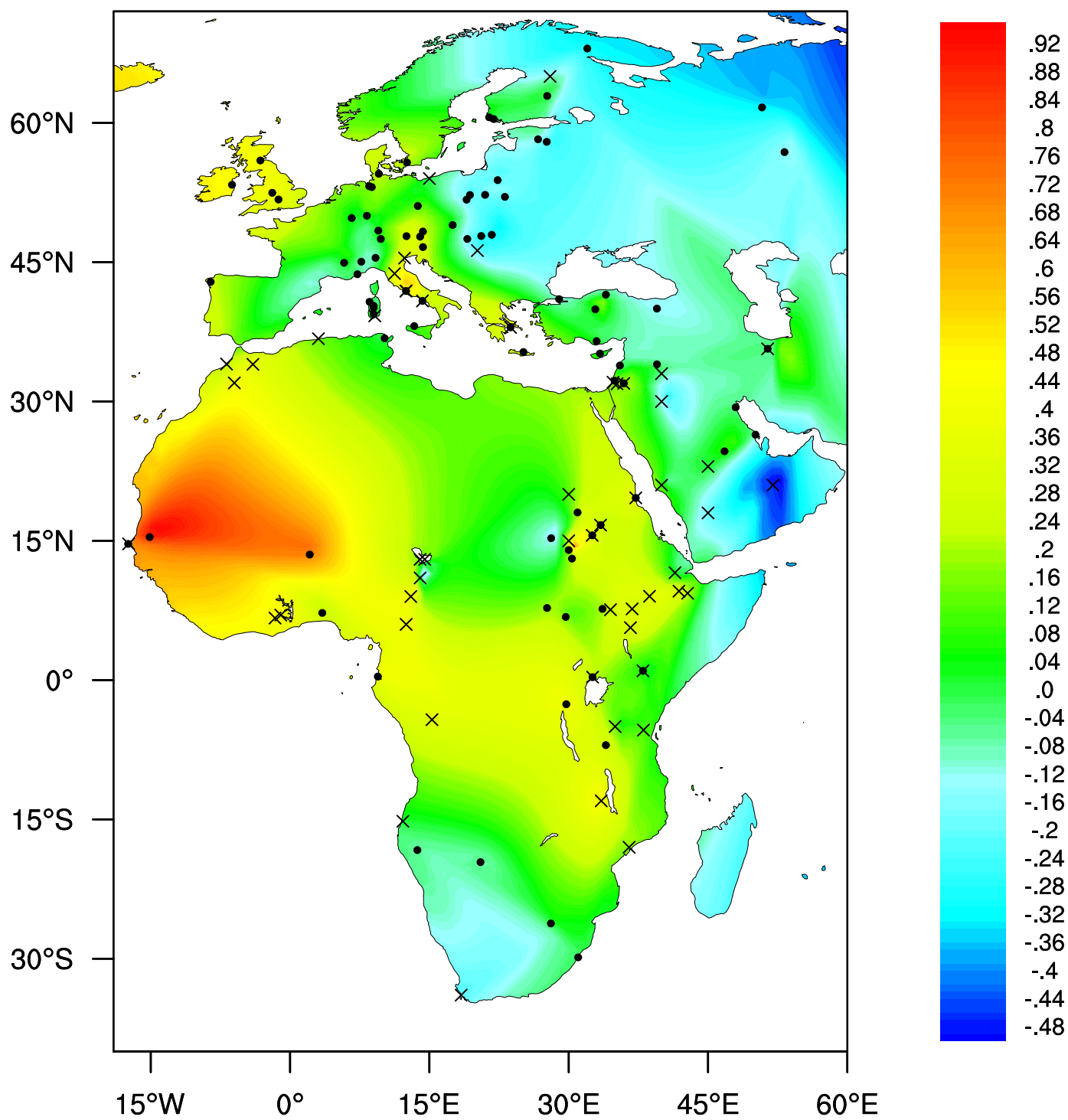

Supplement: Additional file 6 — Africa and Middle East LP genotype-phenotype correlation, obtained by calculating the quantitative difference between observed phenotype frequency and predicted phenotype frequency based on locations where only fully sequenced data of all 4-LP associated alleles was available. Positive and negative values represent cases of LP-correlated genotype under- and over-predicting the LP phenotype, respectively. Dots represent LP phenotype collection locations, crosses represent data collection locations for all currently known 4 LP-correlated alleles. Colour key shows the values of the predicted LP phenotype frequencies (Figure 4) subtracted from the observed LP phenotype frequencies (Figure 1). The Asia-Pacific data was not analysed since 4 alleles data in these regions is very sparse, and fully sequenced data for western and northern Europe is also sparse. [file 1471-2148-10-36-S6.PDF]

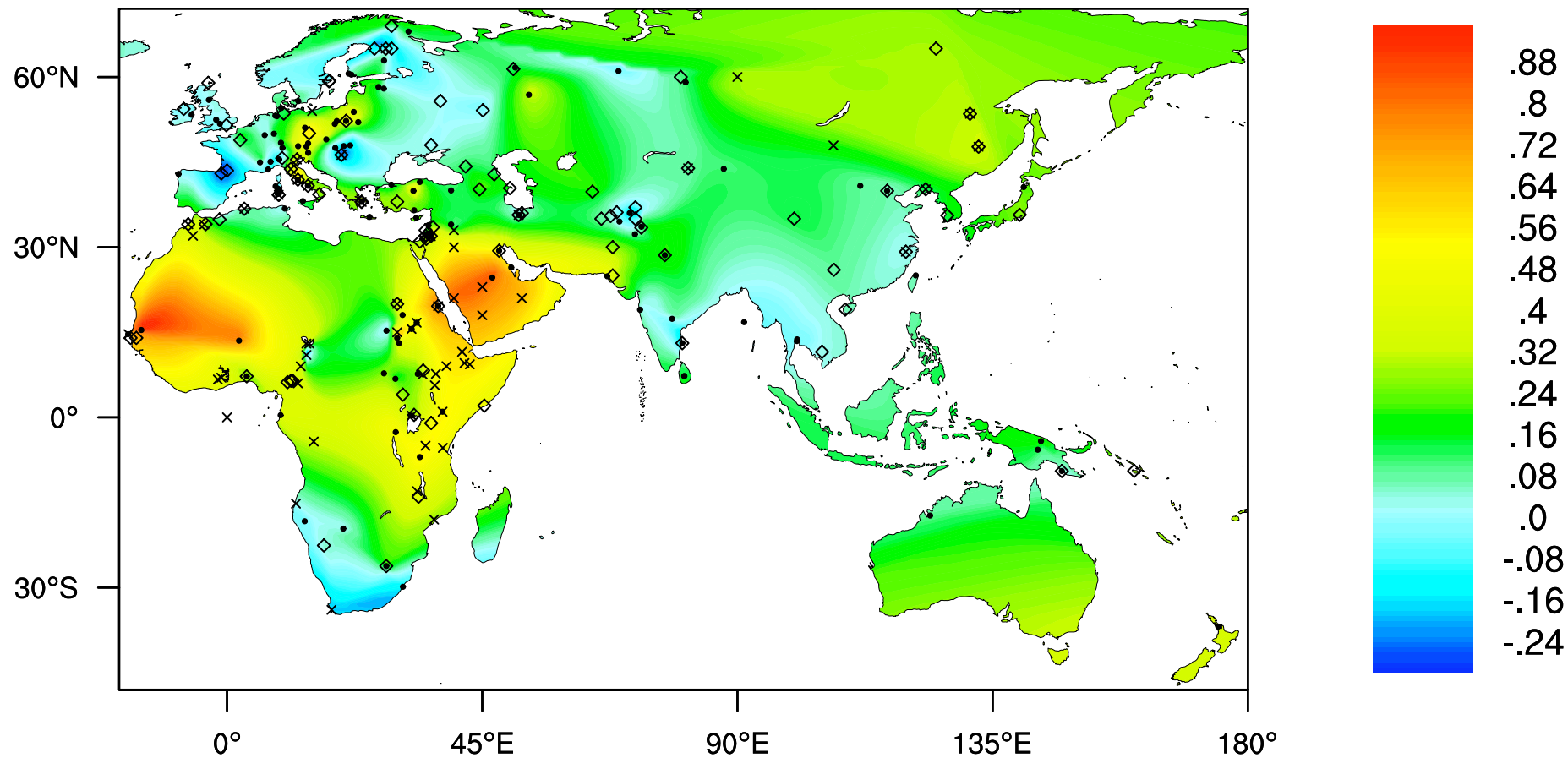

Supplement: Additional file 7 — Old World LP genotype-phenotype correlation, obtained by calculating the quantitative difference between observed phenotype frequency and predicted phenotype frequency based on -13,910*T allele data only. Positive and negative values represent cases of LP-correlated genotype under- and over-predicting the LP phenotype, respectively. Dots represent LP phenotype collection locations, crosses represent data collection locations for the 13,910*T allele obtained from fully sequenced data, and diamonds represent -13,910 C>T only data collection locations. Colour key shows the values of the predicted LP phenotype frequencies predicted by -13,910*T allele data only subtracted from the observed LP phenotype frequencies. [file 1471-2148-10-36-S7.PDF]

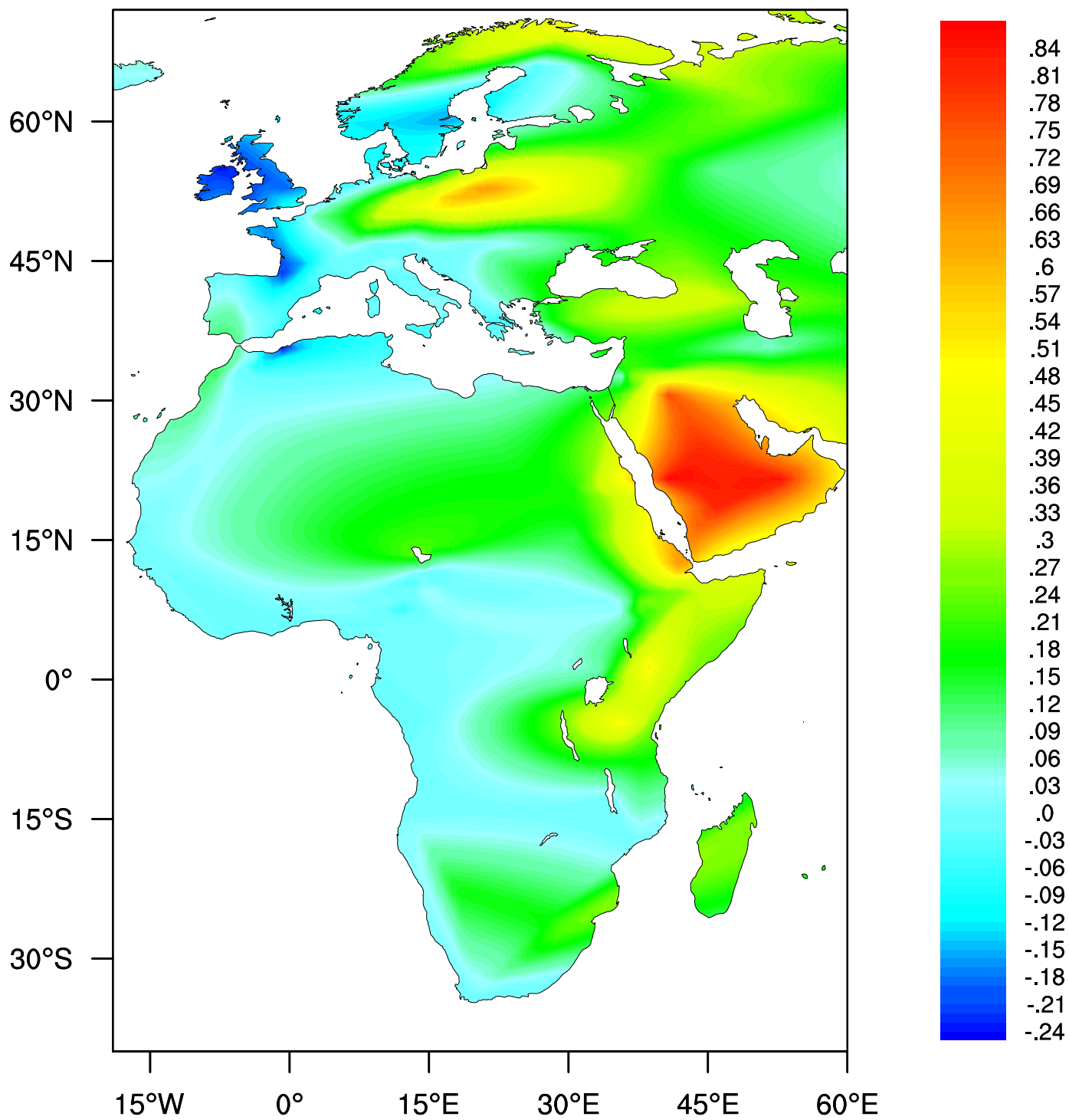

Supplement: Additional file 8 — The difference between the maps of Additional Files 6 and 7, demonstrating the additional knowledge acquired by the 3 additional LP-associated alleles (other than the -13,910*T allele). The Asia-Pacific data was not analysed since 4 alleles data in these regions is very sparse. [file 1471-2148-10-36-S8.PDF]
